# Chinese Herbal Medicine (*Xiaoaiping*) Injections for Chemotherapy-Induced Thrombocytopenia: A Randomized, Controlled, Multicenter Clinical Trial

**DOI:** 10.1089/acm.2018.0470

**Published:** 2019-06-04

**Authors:** Shuo Qi, Xiao Li, Qing Dong, Hezheng Lai, Dianna Porter, Shaodan Tian, Li Hou, Xinyi Chen, Xiaoke Li, Kang Wang

**Affiliations:** ^1^ Dongzhimen Hospital, Affiliated to Beijing University of Chinese Medicine (BUCM), Beijing, China.; ^2^ School of Science and Health, Western Sydney University, Campbelltown, Australia.; ^3^ Chinese Medicine Center, Western Sydney University, Campbelltown, Australia.; ^4^ NICM Health Research Institute, Westmead Campus, Western Sydney University, Penrith, Australia.; ^5^ Dongfang Hospital, Affiliated to Beijing University of Chinese Medicine (BUCM), Beijing, China.

**Keywords:** neoplasm, Traditional Chinese Medicine, Chinese herbal medicine, chemotherapy-induced thrombocytopenia

## Abstract

***Objectives:*** The study aims to evaluate the therapeutic efficacy and safety of Chinese herbal medicine (*Xiaoaiping*) injections for chemotherapy-induced thrombocytopenia (CIT) in nonsmall cell lung cancer (NSCLC) and gastric cancer.

***Design:*** A randomized, controlled, multicenter study from December 2013 to August 2015.

***Settings/Location:*** All patients are from China.

***Subjects:*** One hundred forty patients with either NSCLC or gastric cancer were enrolled in this trial.

***Interventions:*** The intervention group (*n* = 70) was given *Xiaoaiping* injections (1 dose/day for 10 days) with chemotherapy, whereas the control group (*n* = 70) was given chemotherapy only. The follow up period was 11 days after the final injection.

***Outcome measures:*** Platelet (PLT) count was tested at day 0, 7, 14, and 21 as the primary outcome for evaluation. Safety measurements, including red blood cells (RBC), hemoglobin (HBG), white blood cells (WBC), neutrophil (NE)#, aspartate aminotransferase (AST), alanine aminotransferase (ALT), lactate dehydrogenase (LDH), creatine kinase (CK), creatinine (Cr), and blood urea nitrogen (BUN) were tested at day 0 and 21 as the secondary outcomes.

***Results:*** (1) Two patients in the intervention group and four patients in the control group were lost upon follow-up. (2) PLT count: there was no significant difference in PLT count between the two groups from baseline (day 0), day 7, and day 14. At day 21, the intervention group indicated an upward trend of PLT count with a statistically significant difference than that of the control group (*p* < 0.05). (3) NSCLC: there was significant difference in PLT count between the two groups on day 21 (*p* < 0.01). (4) Gastric cancer: there was no significant difference in PLT count between the two groups during this trial (*p* > 0.05). (5) There was no statistically significant difference between the intervention group and the control group with the safety figures (secondary outcomes) RBC, HGB, WBC, NE#, AST, ALT, LDH, CK, Cr, and BUN measured (*p* > 0.05). (6) Adverse events: one gastric cancer patient in the control group was diagnosed with gastrointestinal bleeding on day 3.

***Conclusions:*** In conclusion, *Xiaoaiping* injections may provide a safe and effective option for CIT in patients with NSCLC.

## Introduction

Cancer is the leading cause of death in China and a major public health concern, increasing in rate of both incidence and mortality.^1–3^ Lung cancer (incidence 733.3/100,000 and mortality 610.2/100,000) and gastric cancer (incidence 679.1/100,000 and mortality 498.0/100,000) are the first and second most prominent cancers in China, respectively, in terms of incidence and mortality rates.^4^

Chemotherapy is the predominant treatment method for cancer with substantial progress being made in the last 50 years. Platinum-based chemotherapy is used significantly, with benefits, including increasing overall survival rates, enhancing quality of life (QoL), and increasing survival in advanced-stage disease.^5^ Unfortunately, thrombocytopenia is a common side effect in cancer patients resulting from cytotoxicity.^6^ Development of chemotherapy-induced thrombocytopenia (CIT) may cause a delay or reduction to the course of chemotherapy, subsequently affecting its effectiveness.^7^ Moreover, CIT is the main dose-limiting toxicity factor of platinum-based chemotherapy.^8^ CIT is treated with platelet (PLT) transfusion, recombinant human thrombopoietin (rhTPO), and recombinant human interleukin-11 (rhIL-11),^9^ all of which are approved by the China Food and Drug Agency (CFDA). There are, however, present risks associated with transfusion in terms of transfusion-related acute lung injuries.^10^ Several side effects of rhTPO and rhIL-11 have been documented, including fever, chills, fatigue, rash, edema, dyspnea, pleural effusion, atrial arrhythmia, and headache.^11,12^

Traditional Chinese Medicine (TCM) is a complementary and alternative medicine that is used not only in the treatment of cancer and cancer-related conditions, but also to improve the side effects of anticancer therapies.^13^ A meta-analysis of clinical studies has found that TCM may improve cancer-related symptoms, QoL, enhance immune function and reduce the side effects of chemotherapy.^14^

TCM theory dictates the pathology of CIT as a heat toxicity that damages the *qi* and *yin*, and herbs functioning to clear heat toxins may thereby remedy or prevent CIT. *Xiaoaiping* injection is a traditional Chinese herbal medicine extracted from the root of *Marsdenia tenacissima* (*Tong Guang Teng*), which is cooling in nature.^15^ Clinical research indicates that *Xiaoaiping* injections not only significantly inhibit the growth of gastric cancer cells, but also improve the effectiveness of chemotherapy, improve QoL, and reduce the incidence of bone marrow suppression when used during chemotherapy.^16–20^ In addition, a retrospective study found that *Xiaoaiping* injections have the function of improving CIT in advanced nonsmall cell lung cancer (NSCLC) patients.^21^ To further evaluate the effectiveness and safety of *Xiaoaiping* injections for CIT, a randomized, controlled, multicenter clinical trial was performed from December 2013 to August 2015.

## Materials and Methods

### Study design

The study is a randomized, controlled, multicenter trial. It is registered in the Chinese Clinical Trial Registry (ChiCTR) under registration no.: ChiCTR-TRC-13003888.

A study sample size of 120 patients was calculated based upon a prior study.^21^ The allocation ratio was 1:1, with 60 patients in the intervention group and 60 patients in the control group. A sample size of 144 patients was deemed sufficient to compensate for a 20% attrition rate.^21^

Allocation: independent third-party researchers used opaque envelopes to seal distribution cards containing computer-generated random numbers that were then sent to researchers at collaborating centers. Once a patient was deemed eligible, they were randomized into a group according to the provision on the distribution card. Patients and researchers were not blinded to the type of treatment, and the participants were informed of the study design when recruited.

### Participants

The participants were recruited from six collaborating medical hospitals in China.

#### Inclusion criteria

Inclusion criteria: (1) diagnosed with NSCLC or gastric cancer by imaging and/or histopathology or cytology; (2) no age limit; (3) Karnofsky Performance Status score ≥60; (4) Expected survival time ≥6 months; (5) indicated to receive initial platinum-based chemotherapy only, without receiving radiotherapy and/or chemotherapy within 2 months of trial commencement; (6) no diagnosis of comorbid disease (heart, liver, kidney, blood disease); and (7) willingness to participate in clinical research and to sign informed consent.

#### Exclusion criteria

Exclusion criteria: (1) did not meet the inclusion criteria; (2) mental disorders (cancer combined with moderate depression or above) as assessed by previous medical history or referring clinician (to exclude patients unable to correctly describe the subjective symptoms); (3) patients who had serious uncontrolled comorbid disease or acute infection; (4) pregnant or lactating women; (5) previous record of poor drug compliance by medical treatment history.

### Treatment and follow-up

Patients in both groups received conventional chemotherapy. The intervention group received a daily *Xiaoaiping* injection for 10 consecutive days beginning on the first day of chemotherapy (approval no.: Z20025868; Manufacturer: Nanjing Sanhome Pharmaceutical Co., Ltd.; Specifications: 3 × 20 mL/dose). The patients in the control group were given conventional chemotherapy only. The trial involved 10 days of treatment with an 11-day follow-up period.

### Outcome measures

#### Primary outcomes measurements

Primary outcomes: effectiveness was measured with PLT count of both groups. PLT count was assessed at the baseline and at three time points: day 7, 14, and 21. The data of each time point was analyzed and compared between intervention and control groups, and for both NSCLC and gastric cancer subgroup patients.

#### Secondary outcome measurements

Secondary outcomes: safety measurements included count of white blood cells (WBC), red blood cells (RBC), hemoglobin (HGB), alanine aminotransferase (ALT), aspartate aminotransferase (AST), lactate dehydrogenase (LDH), creatine kinase (CK), blood urea nitrogen (BUN), and creatinine (Cr). The above figures were assessed at baseline and day 21. The data from each time point was analyzed between the two groups.

#### Adverse events

All participants were asked about any adverse events during their visits and were also instructed to record any unexpected events throughout the trial in a diary.

### Statistics methods

The analyses were performed using SPSS software (IBM, Version 21.0) by a third-party clinical evaluation center. Frequencies and descriptive statistics were used for patients' demographic presentation, mean and standard deviations were calculated for the continuous variables, and group differences were analyzed using Pearson chi-square test for categorical variables. A Kolmogorov–Smirnov test was used to check the distribution of data. Independent *t*-tests were used for normally distributed data, while Mann–Whitney *U* tests were used for non-normal distributions. The primary outcome of PLT count in both groups, PLT count in NSCLC subgroup, PLT in gastric cancer subgroup, and secondary outcomes were analyzed using nonparametric tests. The significance level was set to *p* < 0.05 for all analyses.

## Results

### Characteristics of the patients

**FIG. 1.**
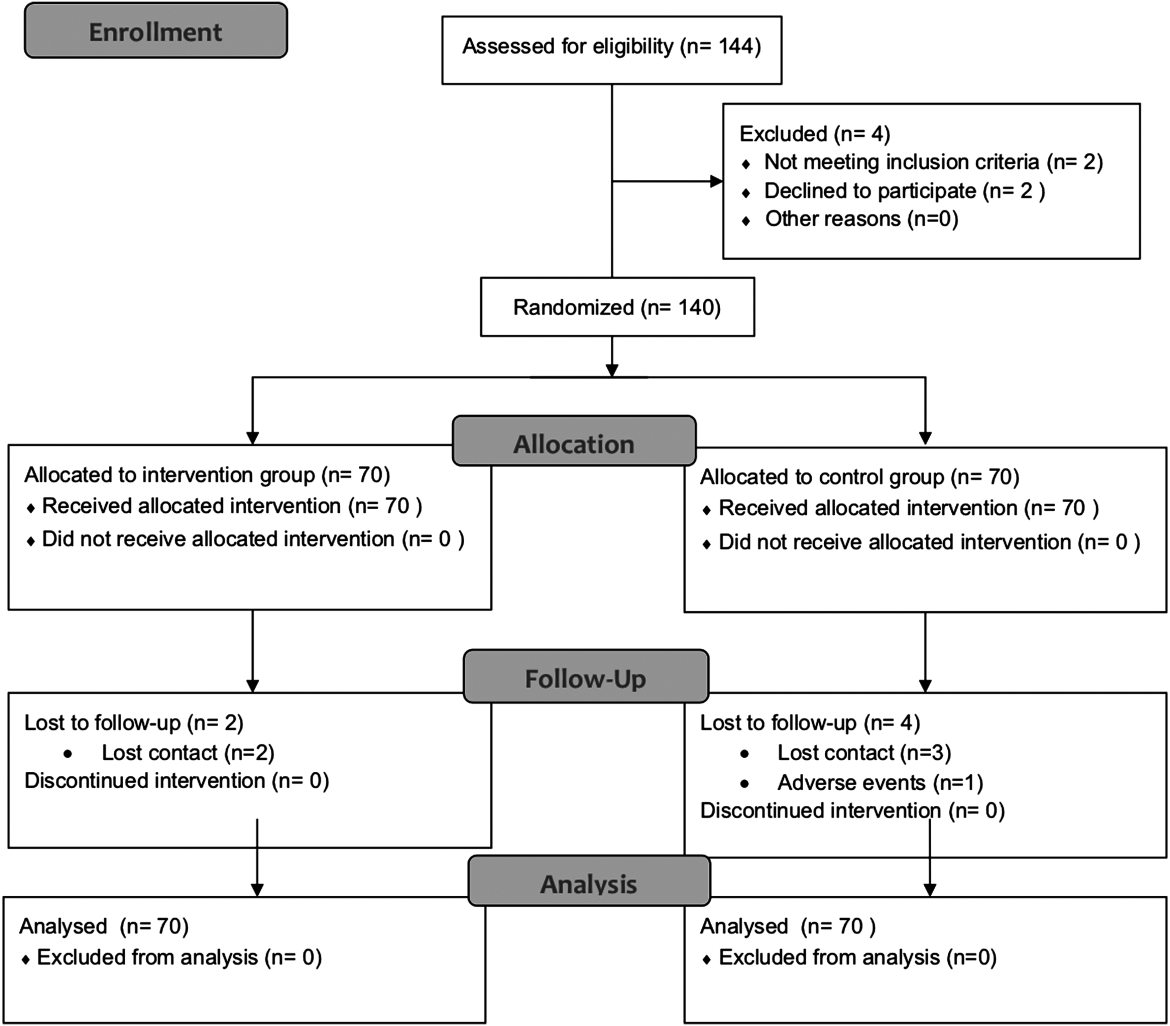
Flow diagram of enrolment.

From December 2013 to August 2015, 144 patients were monitored in total, with 140 patients allocated evenly between an intervention group (*n* = 70) and a control group (*n* = 70) from six collaborating medical centers. A total of two patients in the intervention group and four patients in the control group were lost to follow-up. The data of patients lost is included in the full analysis set. The study protocol followed the recommendations outlined in the Consolidated Standards of Reporting Trials (CONSORT^22^) (Fig. 1).

There were no significant differences at the baseline between intervention and control groups' patient characteristics, in terms of age, gender ratio, and cancer type ratio (Table 1).

**Table 1.** Comparison of Baseline Characteristics Between the Two Groups

*Variable*	*Intervention group (*n* = 70)*	*Control group (*n* = 70)*	p
Age (mean ± SD)	63.23 ± 7.83 (95% CI: 61.36–65.30)	63.50 ± 10.41 (95% CI: 61.02–65.98)	0.60
Gender (*n*/%)			
Male	51 (72.86)	51 (72.86)	>0.05
Female	19 (27.14)	19 (27.14)	
Cancer type (*n*/%)			
Lung cancer	46 (65.71)	46 (65.71)	>0.05
Gastric cancer	24 (34.29)	24 (34.29)	

CI, confidence interval; SD, standard deviation.

### Primary outcomes

**FIG. 2.**
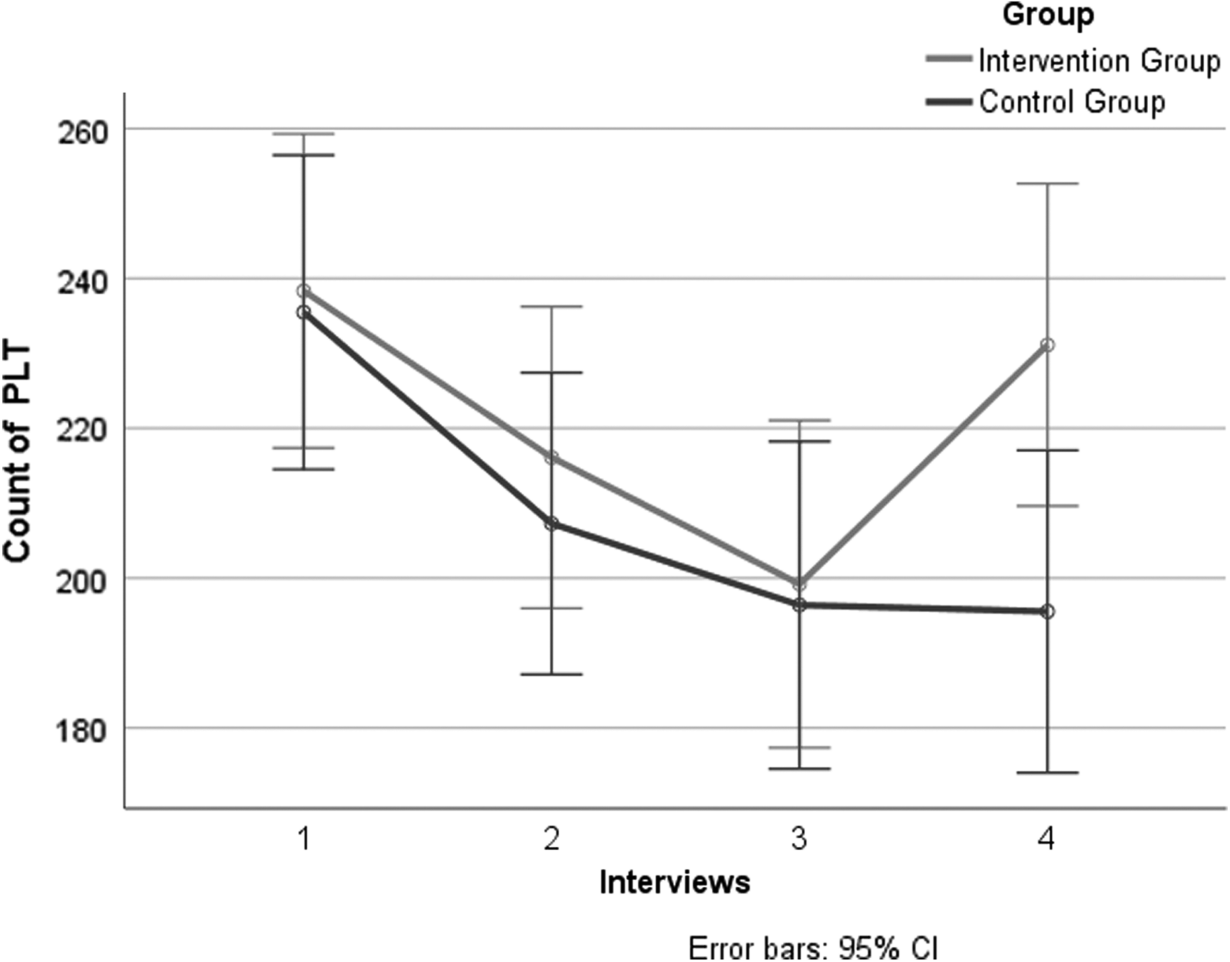
Primary outcomes of PLT count in the two groups of four interviews. CI, confidence interval; PLT, platelet.

During the period of treatment and subsequent follow-up, there was no significant difference in PLT count between the two groups at baseline, day 7, and 14. There was significant downward trend in PLT count from baseline to day 21 in both groups. At day 21 the control group showed a continuing downward trend of PLT count, while the intervention group showed an upward trend of PLT count; there was a statistically significant difference between the two groups at this time (*p* < 0.05). (Table 2; Fig. 2)

**Table 2.** Primary Outcomes of Platelet Count in Two Groups

	*Intervention group (*n* = 70)*		*Control group (*n* = 70)*		p
	*Mean ± SD*	*95% CI*	*Mean ± SD*	*95% CI*	
Day 0	238.31 ± 91.18	216.57–260.05	235.47 ± 86.13	214.93–256.01	0.85
Day 7	216.10 ± 89.52	194.76–237.44	207.27 ± 80.63	226.50–288.05	0.61
Day 14	199.20 ± 92.00	177.26–221.14	196.40 ± 92.84	174.26–218.54	0.89
Day 21	231.13 ± 91.94	209.21–253.05	195.54 ± 90.08	174.06–217.02	0.02^*^

^*^ *p* < 0.05 comparison between two groups.

CI, confidence interval; SD, standard deviation.

**FIG. 3.**
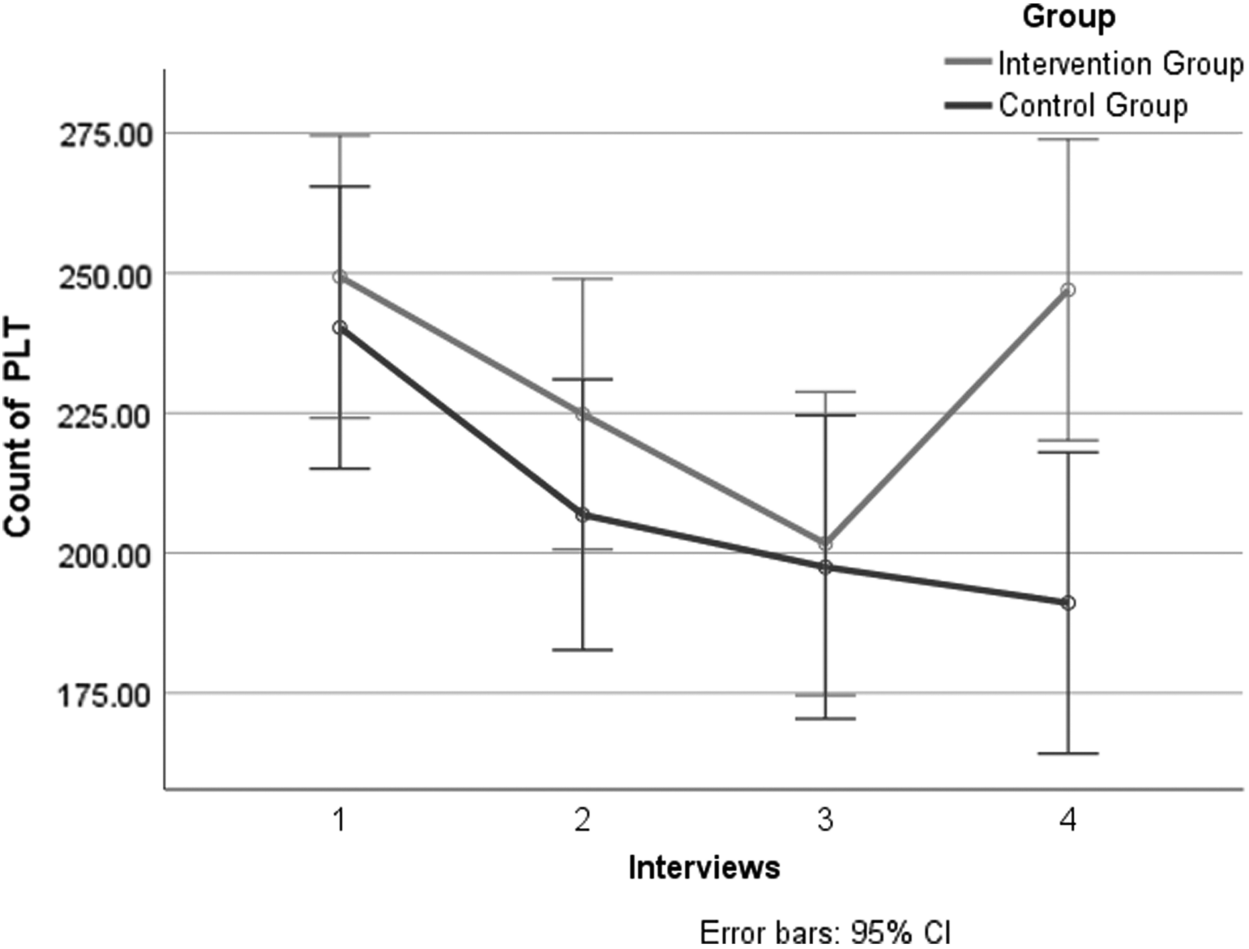
Primary outcomes: subgroup analysis of PLT count in NSCLC patients of four interviews. CI, confidence interval; NSCLC, nonsmall cell lung cancer; PLT, platelet.

Further subgroup analysis of NSCLC patients: PLT count in NSCLC patients in the two groups decreased from baseline to day 21. A significant difference existed between the two groups (*p* < 0.01) (Table 3; Fig. 3).

**Table 3.** Primary Outcomes: Subgroup Analysis of Platelet Count in Nonsmall Cell Lung Cancer Patients

	*Intervention group (*n* = 46)*		*Control group (*n* = 46)*		p
	*Mean ± SD*	*95% CI*	*Mean ± SD*	*95% CI*	
Day 0	249.35 ± 94.24	221.36–277.34	240.26 ± 76.97	217.40–263.12	0.61
Day 7	224.80 ± 91.05	197.77–251.84	206.85 ± 72.93	185.19–228.51	0.42
Day 14	201.67 ± 93.62	173.87–229.47	197.48 ± 91.39	170.34–224.62	0.75
Day 21	247.00 ± 92.15	219.63–274.37	191.09 ± 91.47	163.92–218.25	0.004^**^

^**^ *p* < 0.01 comparison between two groups.

CI, confidence interval; SD, standard deviation.

**FIG. 4.**
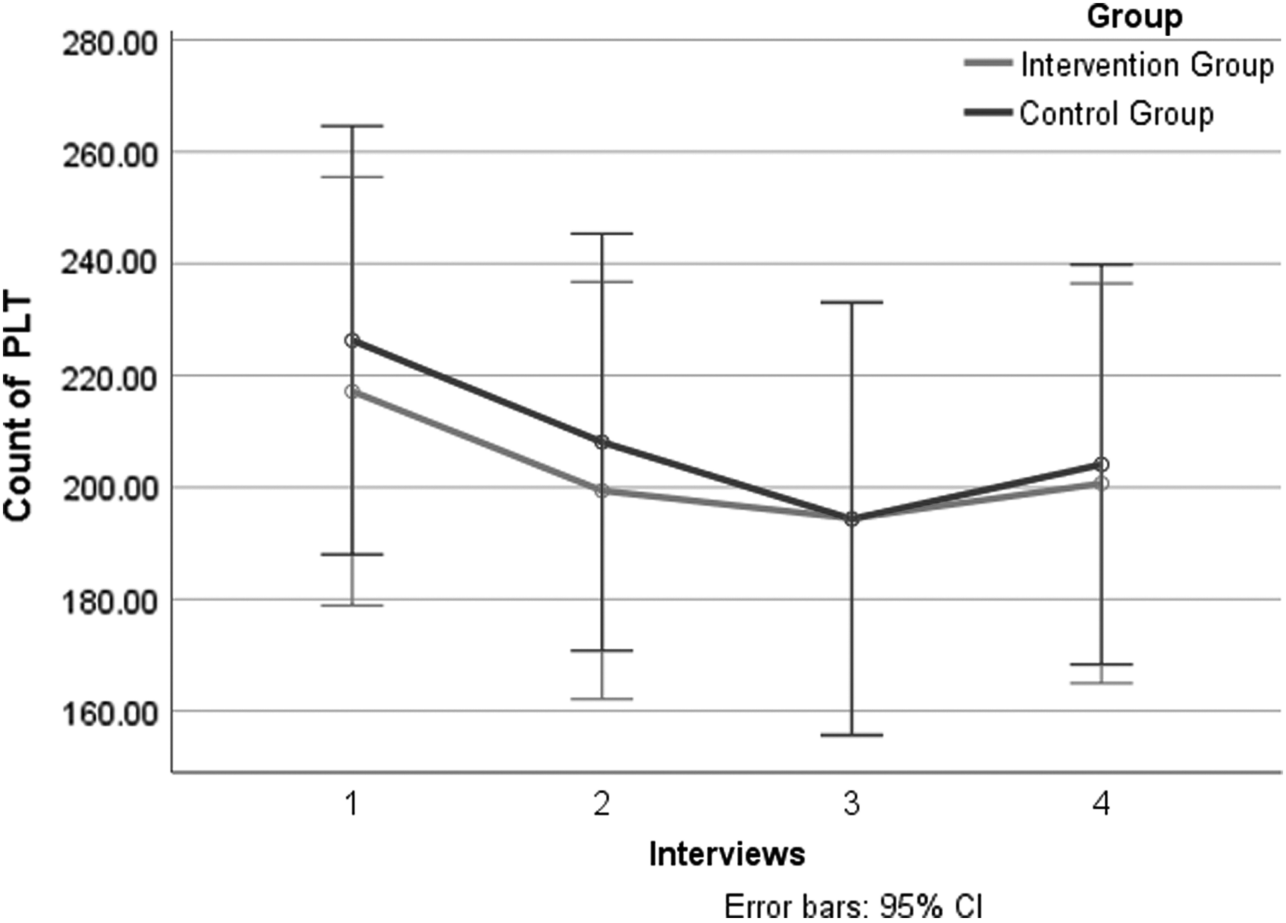
Primary outcomes: subgroup analysis of PLT count in gastric cancer patients of four interviews. CI, confidence interval; PLT, platelet.

Further subgroup analysis of gastric cancer patients: PLT count decreased in gastric cancer patients and there was no significant difference between the two groups (Table 4; Fig. 4).

**Table 4.** Primary Outcomes: Subgroup Analysis of Platelet Count in Gastric Cancer Patients

	*Intervention group (*n* = 24)*		*Control group (*n* = 24)*		p
	*Mean ± SD*	*95% CI*	*Mean ± SD*	*95% CI*	
Day 0	217.17 ± 82.78	182.21–252.12	226.29 ± 102.63	182.96–269.63	0.74
Day 7	199.42 ± 85.89	163.15–235.69	208.08 ± 95.36	167.82–248.35	0.83
Day 14	194.46 ± 90.62	156.19–232.72	194.33 ± 97.52	153.15–235.51	0.94
Day 21	200.71 ± 85.30	164.69–236.73	204.08 ± 88.64	166.65–241.51	0.74

CI, confidence interval; SD, standard deviation.

### Secondary outcomes

There was no statistically significant difference between the intervention group and the control group with safety figures, in terms of RBC, HGB, WBC, neutrophil (NE)#, AST, ALT, LDH, CK, Cr, and BUN (*p* > 0.05) (Table 5).

**Table 5.** Secondary Outcomes of Safety Figures in Two Groups from Day 0 and 21

	*Intervention group (*n* = 70)*		*Control group (*n* = 70)*		p
	*Mean ± SD*	*95% CI*	*Mean ± SD*	*95% CI*	
Day 0					
RBC	3.96 ± 0.58	3.82–4.09	3.91 ± 0.77	3.73–4.09	0.70
HGB	118.04 ± 21.18	112.99–123.09	114.31 ± 27.11	107.85–120.78	0.37
WBC	7.07 ± 3.04	6.35–7.80	6.93 ± 2.77	6.27–7.59	0.95
NE#	4.76 ± 2.84	4.08–5.44	4.65 ± 2.63	4.02–5.28	0.91
AST	20.98 ± 10.43	18.50–23.47	22.94 ± 17.96	18.66–27.23	0.49
ALT	20.22 ± 15.82	16.45–24.00	21.01 ± 19.04	16.47–25.55	0.95
LDH	221.81 ± 143.36	187.62–255.99	199.45 ± 61.21	184.86–214.05	0.70
CK	53.42 ± 28.40	46.65–60.19	54.36 ± 27.45	47.81–60.90	0.78
Cr	58.52 ± 14.63	55.03–62.01	67.36 ± 36.11	58.77–75.98	0.23
BUN	6.78 ± 8.95	4.64–8.91	5.96 ± 4.62	4.86–7.06	0.51
Day 21					
RBC	3.82 ± 0.68	3.66–3.99	3.69 ± 0.64	3.54–3.84	0.22
HGB	115.78 ± 19.62	111.10–120.45	110.51 ± 19.41	105.88–115.14	.11
WBC	6.66 ± 4.17	5.67–7.66	6.56 ± 2.61	5.94–7.19	.27
NE#	5.21 ± 8.76	3.12–7.30	4.44 ± 2.35	3.88–5.00	.13
AST	23.21 ± 10.71	20.66–25.77	27.84 ± 22.36	22.51–33.18	.32
ALT	21.36 ± 13.29	18.19–24.53	28.42 ± 55.42	15.21–41.64	.65
LDH	210.89 ± 108.62	184.99–236.79	179.34 ± 67.28	163.30–195.39	.21
CK	59.68 ± 33.91	51.59–67.76	51.58 ± 19.06	47.04–56.13	.15
Cr	56.51 ± 13.58	53.27–59.75	59.12 ± 25.20	53.11–65.13	.79
BUN	5.33 ± 2.04	4.85–5.82	6.15 ± 5.65	4.80–7.50	.98

ALT, alanine aminotransferase; AST, aspartate aminotransferase; BUN, blood urea nitrogen; CI, confidence interval; CK, creatine kinase; Cr, creatinine; HGB, hemoglobin; LDH, lactate dehydrogenase; NE, neutrophil; RBC, red blood cells; SD, standard deviation; WBC, white blood cells.

### Adverse events

During the study, one gastric cancer patient in the control group was diagnosed with gastrointestinal bleeding on day 3 and was withdrawn from the study.

## Discussion

CIT is a toxic side effect of platinum-based chemotherapeutics.^23^ In the rationale of TCM, chemotherapy drugs generate heat toxins, which directly damage bone marrow and blood collaterals leading to marrow collateral damage and obstructed blood movement. Stagnation of toxic pathogens in bone marrow and weakened generation and transformation of bone marrow cause *qi-blood* depletion. Blood stasis caused by obstruction of movement inhibits the generation of new blood, and as a result, lack of moistening and nourishing leads to deficiency and damage of the bone marrow. Furthermore, the deficiency and damage of bone marrow worsens blood stasis, forming a negative feedback loop. According to “Treatise on Blood Syndromes” (*Xue Zheng Lun*), “Blood stasis in the human body cannot be added to the good blood, instead it inhibits the generation and transformation of new blood. As a result, patients with blood stasis should be treated with the principle of removing stasis.”

TCM is a prominent complementary and alternative medicine therapy used in oncology.^4,13^ A systematic review^18^ reported that Chinese medicine injections are commonly used to relieve chemotherapy-related symptoms, such as leukopenia, vomiting, and nausea, with *Xiaoaiping* being used most frequently. It is a patented, single-herb Chinese medicine extracted from the roots of *M. tenacissima* with primary constituents, including C_21_ steroidal saponins, organic acids, triterpenes, and polysaccharides.^24^ Studies indicate that *Xiaoaiping* encourages the infiltration and function of CD8+ T cells, which then boosts the antigrowth effects of cisplatin on Lewis lung cancer cell xenografts,^25^ it inhibits angiogenesis by downregulation of vascular endothelial growth factor signaling and protein kinase B pathway in cancer cells,^26^ and retrospective research suggests it may improve CIT.^21^ On this basis, a randomized, controlled, multicenter trial was designed. The study demonstrated that there was no significant difference in PLT count at day 7 and 14 (*p* > 0.05), but PLT count in the intervention group was higher than in the control group at day 21 with a significant difference (*p* < 0.05). Further analysis found that there was no significant difference in PLT count among gastric cancer patients in the two groups regardless of time point. However, a statistically significant difference was found in the NSCLC group at day 21 (*p* < 0.01).

The determination of the safety of Chinese medicine for cancer care remains a priority.^27^ RBC, HGB, WBC, NE#, AST, ALT, LDH, CK, Cr, and BUN were tested in this study with no significant differences between the two groups at the baseline and day 21 (*p* > 0.05). No severe adverse events occurred in the intervention group.

## Conclusions

In conclusion, *Xiaoaiping* injections may provide a safe and effective option for CIT in patients with NSCLC. The strength of the trial lies in its novelty of being the first randomized, controlled, multicenter trial focusing on Chinese herbal medicine injections for CIT, with promising results. However, future trials are advised due to several concerns: a possibility exists of performance bias because researchers, patients, and data analysts were not blinded; the follow-up time was limited; and multiple chemotherapy regimens were included, which may affect the accuracy of our results.

## References

[1] Chen W, Zheng R, Zeng H, et al. Annual report on status of cancer in China, 2011. Chin J Cancer Res 2015;27:2–12 25717220 10.3978/j.issn.1000-9604.2015.01.06 PMC4329176

[2] Chen W, Zheng R, Zuo T, et al. National cancer incidence and mortality in China, 2012. Chin J Cancer Res 2016;28:1–11 27041922 10.3978/j.issn.1000-9604.2016.02.08 PMC4779764

[3] Chen WQ, Li H, Sun KX, et al. Report of Cancer Incidence and Mortality in China, 2014 [In Chinese]. Zhonghua zhong liu za zhi [Chin J Oncol] 2018;40:5–13 10.3760/cma.j.issn.0253-3766.2018.01.002 29365411

[4] Chen W, Zheng R, Baade PD, et al. Cancer statistics in China, 2015. CA Cancer J Clin 2016;66:115–132 26808342 10.3322/caac.21338

[5] Wakelee H, Kelly K, Edelman MJ 50 Years of progress in the systemic therapy of non-small cell lung cancer. American Society of Clinical Oncology educational book American Society of Clinical Oncology Meeting 2014:177–189 10.14694/EdBook_AM.2014.34.177 PMC5600272 24857075

[6] Kuter DJ Managing thrombocytopenia associated with cancer chemotherapy. Oncology (Williston Park, NY) 2015;29:282–294 25952492

[7] Parameswaran R, Lunning M, Mantha S, et al. Romiplostim for management of chemotherapy-induced thrombocytopenia. Support Care Cancer 2014;22:1217–1222 24414994 10.1007/s00520-013-2074-2

[8] Yamaguchi K, Kusaba H, Makiyama A, et al. The risk factors for oxaliplatin-induced peripheral sensory neuropathy and thrombocytopenia in advanced gastric cancer. Cancer Chemother Pharmacol 2018 DOI: 10.1007/s00280-018-3652-2 30043209

[9] Chinese Society of Clinical Onocology. Expert consensus on diagnosis and treatment of chemotherapy-induced thrombocytopenia in cancer patients (2014 version) [In Chinese]. Zhonghua zhong liu za zhi [Chin J Oncol] 2014;36:876–879 25620489

[10] Dunbar NM Current options for transfusion-related acute lung injury risk mitigation in platelet transfusions. Curr Opin Hematol 2015;22:554–558 26390161 10.1097/MOH.0000000000000187

[11] Bai CM, Xu GX, Zhao YQ, et al. A multi-center clinical trial of recombinant human thrombopoietin in the treatment of chemotherapy-induced thrombocytopenia in patients with solid tumor [In Chinese]. Zhongguo Yi Xue Ke Xue Yuan Xue Bao 2004;26:437–441 15379272

[12] Smith JW, 2nd. Tolerability and side-effect profile of rhIL-11. Oncology (Williston Park, NY) 2000;14(9 Suppl 8):41–47 11033837

[13] Li X, Yang G, Li X, et al. Traditional Chinese medicine in cancer care: A review of controlled clinical studies published in chinese. PLoS One 2013;8:e60338 23560092 10.1371/journal.pone.0060338 PMC3616129

[14] Zhang D, Zheng J, Ni M, et al. Comparative efficacy and safety of Chinese herbal injections combined with the FOLFOX regimen for treating gastric cancer in China: A network meta-analysis. Oncotarget 2017;8:68873–68889 28978164 10.18632/oncotarget.20320 PMC5620304

[15] Huang Z, Wang Y, Chen J, et al. Effect of Xiaoaiping injection on advanced hepatocellular carcinoma in patients. J Tradit Chin Med 2013;33:34–38 23596809 10.1016/s0254-6272(13)60097-7

[16] Ren J Effect of Xiaoaiping on attenuation and synergism and quality of life of malignant tumor after chemotherapy. Xiandai Zhongxiyi Jiehe Zazhi 2015:2691–2693

[17] He L, Liu D, Liu T, et al. Effect of Xiaoaiping on postoperative immune function in patients with esophageal cancer in different ethnic groups. Jianyan Yixue Linchuang 2015:1774–1775

[18] Zhang D, Wu J, Wang K, et al. Which are the best Chinese herbal injections combined with XELOX regimen for gastric cancer?: A PRISMA-compliant network meta-analysis. Medicine (Baltimore) 2018;97:e0127 29561411 10.1097/MD.0000000000010127 PMC5895335

[19] Li J, Zhsng Y, Han L, et al. Advances in pharmacological effects and adverse reactions of Xiaoaiping. Xiandai Yiyao Weisheng 2017:223–225

[20] Yang Z, Hu C Therapeutic effect of Xiaoaiping injection on elderly patients with advanced non-small cell lung cancer. Anhui Yiyao 2010:1470–1471

[21] Lei Y, Sun P, Hou L, et al. Retrospective study of the effect of Xiaoaiping combined with chemotherapy on peripheral platelets and their parameters in patients with advanced non-small cell lung cancer. Yixue Yanjiu Za Zhi 2014:86–89

[22] Schulz KF, Altman DG, Moher D, et al. CONSORT 2010 statement: Updated guidelines for reporting parallel group randomised trials. BMJ 2010;340:c332 20332509 10.1136/bmj.c332 PMC2844940

[23] Minichmayr I, Nock V, Jaehde U, et al. Thrombocytopenia following high-dose chemotherapy with carboplatin, etoposide and thiotepa in patients with testicular germ cell cancer. Int J Clin Pharmacol Ther 2013;51:74–76 23260006 10.5414/cpp51074

[24] Wang PL, Sun Z, Lv XJ, et al. A homologues prediction strategy for comprehensive screening and characterization of C21 steroids from Xiao-ai-ping injection by using ultra high performance liquid chromatography coupled with high resolution hybrid quadrupole-orbitrap mass spectrometry. J Pharm Biomed Anal 2018;148:80–88 28965048 10.1016/j.jpba.2017.09.024

[25] Li W, Yang Y, Ouyang Z, et al. Xiao-Ai-Ping, a TCM injection, enhances the antigrowth effects of cisplatin on lewis lung cancer cells through promoting the infiltration and function of CD8(+) T lymphocytes. Evid Based Complement Alternat Med 2013;2013:879512 23956781 10.1155/2013/879512 PMC3730189

[26] Wang MJ, Du DY, Fan W, et al. [Effects and mechanisms of Xiao-Ai-Ping injection on angiogenesis]. Yao Xue Xue Bao 2016;51:309–315 29856586

[27] Cheng CW, Fan W, Ko SG, et al. Evidence-based management of herb-drug interaction in cancer chemotherapy. Explore (NY) 2010;6:324–329 20832765 10.1016/j.explore.2010.06.004

